# D‐galactose‐induced cardiac ageing: A review of model establishment and potential interventions

**DOI:** 10.1111/jcmm.17580

**Published:** 2022-10-17

**Authors:** Sui‐sui Wang, Xu Zhang, Ze‐zhi Ke, Xiu‐yun Wen, Wei‐dong Li, Wen‐bin Liu, Xiao‐dong Zhuang, Li‐zhen Liao

**Affiliations:** ^1^ Guangdong Engineering Research Center for Light and Health, Guangzhou Higher Education Mega Center Guangdong Pharmaceutical University Guangzhou China; ^2^ Guangdong Provincial Key Laboratory of Pharmaceutical Bioactive Substances, Guangzhou Higher Education Mega Center Guangdong Pharmaceutical University Guangzhou China; ^3^ Cardiology Department The First Affiliated Hospital of Sun Yat‐Sen University Guangzhou China

**Keywords:** apoptosis, autophagy, cardiac ageing, cardiac function, D‐galactose

## Abstract

Cardiovascular disease (CVD) is highly prevalent in an ageing society. The increased incidence and mortality rates of CVD are global issues endangering human health. There is an urgent requirement for understanding the aetiology and pathogenesis of CVD and developing possible interventions for preventing CVD in ageing hearts. It is necessary to select appropriate models and treatment methods. The D‐galactose‐induced cardiac ageing model possesses the advantages of low mortality, short time and low cost and has been increasingly used in the study of cardiovascular diseases in recent years. Therefore, understanding the latest progress in D‐galactose‐induced cardiac ageing is valuable. This review highlights the recent progress and potential therapeutic interventions used in D‐galactose‐induced cardiac ageing in recent years by providing a comprehensive summary of D‐galactose‐induced cardiac ageing in vivo and in vitro. This review may serve as reference literature for future research on age‐related heart diseases.

## 1 INTRODUCTION

Ageing is widely defined as a time‐dependent functional decline that affects most organisms.^1^ It is characterized by a gradual loss of physiological integrity, resulting in multifaceted structural and functional microcirculation damage, which damages multiple organ functions.^2^, ^3^ With the increasing elderly population, there is an increase in age‐related diseases, among which cardiovascular disease is the leading cause of health damage and death in the elderly people in the world.^4^ The ageing of the heart is closely related to cardiovascular diseases; therefore, it is essential to understand the mechanism of cardiac ageing and to choose a suitable model for ageing research.

Laboratory methods of cardiac ageing, such as natural and induced ageing models, have been used to study cardiovascular diseases.^5^, ^6^, ^7^ The naturally ageing model is most suitable for studying the characteristics of human ageing and the ageing mechanism. However, the attributes of a long feeding cycle, poor health and high mortality limit the broad application of the natural ageing model. Many studies have chosen artificially induced ageing models, such as the D‐galactose‐induced ageing model wherein D‐galactose, a reducing monosaccharide, is continuously injected into animals within a certain period and is bound to cause glucose metabolism disorders in essential organs such as the heart. The concentration of galactose increases in cells, and it is reduced to galactitol by aldose reductase catalysis. The latter accumulates in the cell, resulting in cell swelling, dysfunction, and eventually cell ageing. Alternatively, D‐galactose through another metabolic pathway produces reactive oxygen species (ROS) and advanced glycation end‐products (AGEs), which accelerate the ageing process.^8^ Therefore, D‐galactose‐induced ageing models are suitable for studying cardiac ageing.

**FIGURE 1:**
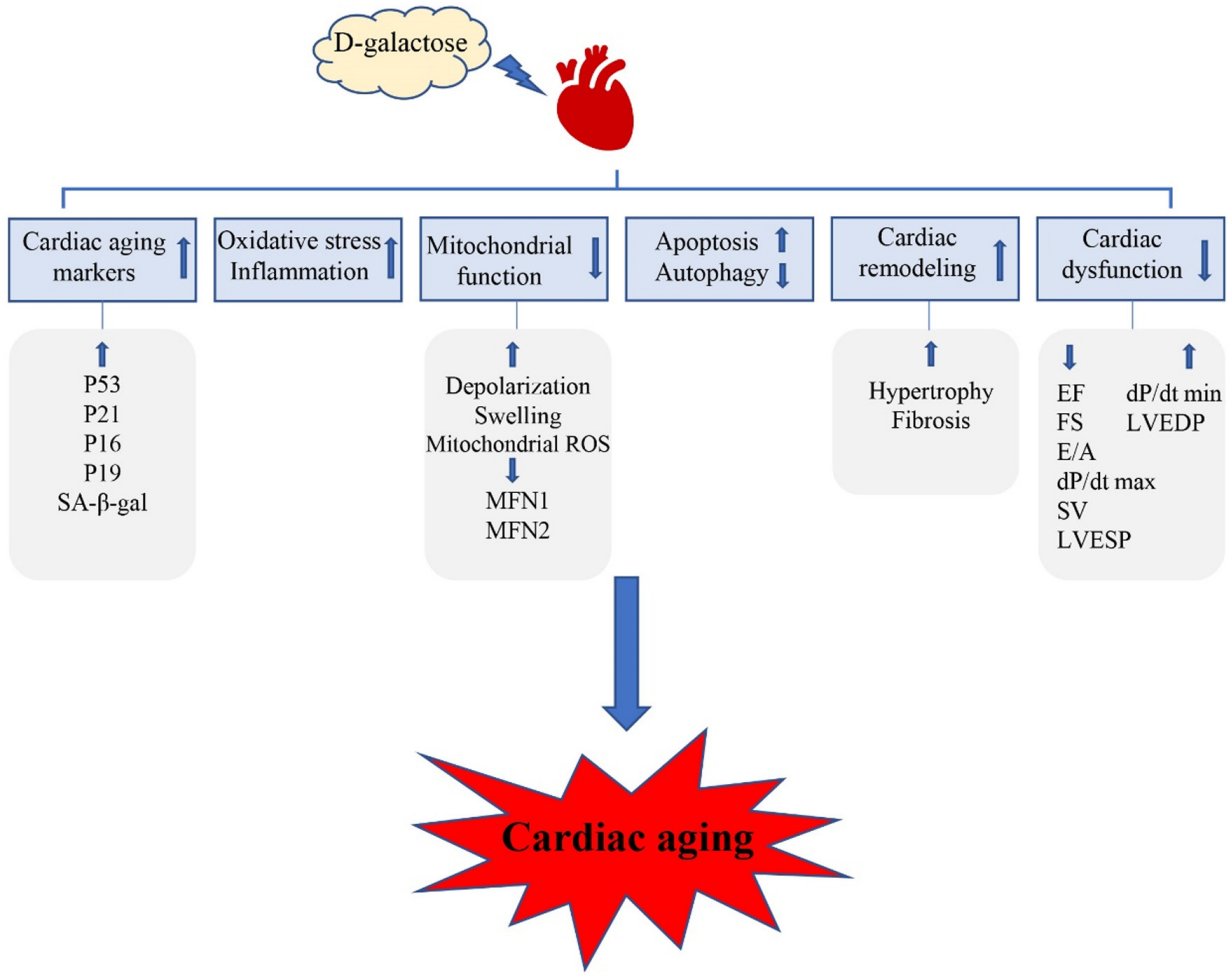
D‐galactose administration induces cardiac ageing. D‐galactose administration increases cardiac ageing markers, oxidative stress, inflammation, apoptosis and cardiac remodelling and reduces mitochondrial function, autophagy and cardiac dysfunction, leading to cardiac ageing

This review comprehensively summarizes studies on D‐galactose‐induced cardiac ageing submitted to PubMed (https://www.ncbi.nlm.nih.gov/PubMed) between January 2017 and March 2022; they were searched in PubMed using the terms ‘D‐galactose and heart’, or’ D‐galactose and cardiac’. Then, the studies related to heart ageing were screened to review literature on the mechanisms of D‐galactose, potential interventions, and changes in related test indicators in cardiac ageing (Figure 1).

## 2 EFFECT OF D‐GALACTOSE ADMINISTRATION ON THE MARKERS OF CARDIAC AGEING AND THE CARDIAC INDEX

Numerous studies have shown that D‐galactose administration increases the markers related to cardiac ageing; they are presented in Table 1. The model utilized 2–3‐month‐old mice and rats injected subcutaneously or intraperitoneally with D‐galactose at a dose of 50–200 mg/kg/day, for 6–10 weeks (Table 1). This protocol significantly increases the levels of senescence‐associated β‐galactosidase (SA‐β‐gal) in the cardiac tissues. During cell senescence, there is increased SA‐β‐gal accumulation and activity due to the expansion and increase in lysosomes. D‐galactose administration significantly increases SA‐β‐gal expression in cardiac tissues.^9^, ^10^, ^11^, ^12^, ^13^ SA‐β‐gal‐positive cells are therefore used as common ageing markers. A series of cell cycle regulatory factors, such as P53, P21, P16 and P19, are also used as ageing markers. As the main participants of the DNA damage response (DDR) path, P53 can trigger a transient cell cycle arrest or permanent cell cycle arrest (cellular senescence).^14^ In response to stimuli that induce a DDR, cell growth arrest and senescence occurs through the MDM2‐P53‐P21 and P16‐pRb pathways.^15^ D‐galactose administration increases the expression of P53, P21, P19 and P16.^9^, ^16^ Telomeres are located at the end of eukaryotic cell chromosomes, comprising a tract of tandemly repeated short DNA repeats and associated protective proteins; telomere attrition is closely related to ageing‐related diseases.^17^ The telomere stability requires cooperation between multiple factors, including telomerase, TRF1, TRF2 and among others.^18^, ^19^, ^20^ Mice treated with D‐galactose for 10 weeks, at a dose of 100–150 mg/kg/day, showed significantly reduced telomere length and TRF1, TRF2 and TERT expressions in cardiac tissues, with this phenomenon being reversed with the use of protective interventional measures.^16^, ^21^

**TABLE 1:** Effect of D‐galactose administration on the markers of cardiac ageing

Ref	Study model	Age	Dose (mg/kg/day)	Route	Duration	Intervention	Major findings	Interpretation
JCR Q2 IF:6.2^9^	C57BL/6	3 months	120	SC	8 weeks	Kanglexin(10 and 20 mg/kg/day); Emodin(20 mg/kg/day); 8 weeks; gavage	SA‐β‐gal staining↑——↓; P53, P21↑——↓	The diastolic dysfunction and cardiac remodelling in mice with D‐gal‐induced ageing were markedly mitigated by KLX and emodin
JCR Q2 IF:7.7^10^	Wistar rats	200–220 g	150	SC	8 weeks	—	SA‐β‐gal staining↑	D‐galactose‐induced ageing further increased cardiac ageing markers in a time‐dependent manner in the presence of an obese insulin‐resistant condition
JCR Q3 IF:3.4^11^	Kunming mice	6–8 weeks	500	SC	60 days	*Polygonatum sibiricum* Polysaccharides(PSP); 200, 400 mg/kg/day; 60 days; Ig	SA‐β‐gal staining↑——↓; P53, P21↑——↓	PSP attenuated D‐gal‐induced cardiac ageing via inhibiting oxidative stress
JCR Q3 IF:5.7^12^	Wistar rats	200–220 g	150	SC	8 weeks (after 12 weeks to induce obese‐insulin resistant condition by eating a high‐fat diet)	Hyperbaric oxygen therapy (HBOT); 100% oxygen (O2) with 250 L/min flow rate; 80 minutes; once daily for 14 days(after 8 weeks d‐gal injection)	SA‐β‐gal staining↑——↓	HBOT effectively alleviates cardiac dysfunction via attenuating mitochondrial dysfunction in pre‐diabetic rats
JCR Q4 IF:2.6^13^	SD rats	8 weeks	150	—	8 weeks	Alpinate Oxyphyllae Fructus (AOF) 50, 100, 150 mg/kg/day; 10 weeks; orally	SA‐β‐gal↑——↓(50, 100↔); p21↑——↓	AOF negatively modulated the D‐galactose‐induced cardiac hypertrophy signalling mechanism to attenuate ventricular hypertrophy
JCR Q2 IF:5^16^	C57BL/6	10‐12 weeks	100	SC	10 weeks	MiR‐21 knockout mice	Telomere length↓——↑; dysregulation of ageing markers (P16INK4a and P19ARF↑——↓; TRF 1, TRF2, and TERT↓——↑)	MiR‐21 knockout had a protective effect against D‐gal‐induced cardiac alterations
JCR Q3 IF:5.7^21^	C57BL/6	8–10 weeks	150	SC	10 weeks	Natural flavone acacetin; 10, 20, 50 mg/kg/day; 10 weeks; Ig	P53, P21↑——↓; Myocardial telomere length↓——↑	Acacetin significantly inhibits *in vivo* cardiac ageing induced by D‐galactose via Sirt1‐mediated activation of Sirt6/AMPK signalling pathway, thereby enhancing mitophagy and preserving mitochondrial function
JCR Q3 IF:2.9^22^	ICR mice	10 weeks	120	IP	6 weeks	Insect tea primary leaf (ITPL); 50 mg/kg, 100 mg/kg; 10 weeks (pretreated 4 weeks and then treated with D‐galactose for 6 weeks); Ig	Cardiac Index↓——↑	ITPL increased superoxide dismutase, glutathione peroxidase, and glutathione levels and reduced nitric oxide and malondialdehyde levels in the serum in oxidative damaged mice induced by D‐gal
JCR Q3 IF:4.4^23^	Kunming mice	6 weeks	120	IP	6 weeks	*Lactobacillus plantarum* CQPC11; 1.0 × 10^9^ CFU/kg; four weeks (3rd week to 6th week); Ig	Cardiac Index↓——↑	LP‐CQPC11 effectively inhibited the reduction in organ indices and alleviated tissue atrophy induced by D‐gal
JCR Q1 IF:4^24^	Kunming mice	10 weeks	120	IP	6 weeks	*Lactobacillus plantarum* KSFY02; 1.0 × 10^9^ CFU/kg; 10 weeks (pretreat 4 weeks and then 6 weeks during the D‐galactose injections); Ig	Cardiac Index↓——↑	LP‐KSFY02 effectively inhibited the decrease in organ indices caused by oxidative ageing and alleviated body tissue atrophy caused by D‐galactose
JCR Q4 IF:2.4^25^	ICR mice	10 weeks	120	IP	6 weeks	*Apocynum venetum* polyphenols(AVP); 50 or 100 mg/kg; 10 weeks (pretreat for 4 weeks and then 6 weeks during the D‐galactose injections); gavage	Cardiac index↓——↑	AVP inhibited the decline of the Cardiac index caused by oxidative stress‐induced tissue atrophy
JCR Q4 IF:2.6^26^	ICR mice	10 weeks	120	IP	6 weeks	Polyphenol extract of small‐leaved Kuding tea (PSLKDT); 50 or 100 mg/kg; 10 weeks (pretreat for 4 weeks and then 6 weeks during the D‐galactose injection ns); gavage	Cardiac index↓——↑	PSLKDT inhibited the decline of the cardiac index caused by oxidative stress‐induced tissue atrophy
JCR Q4 IF:2.6^27^	SD rats	3 months	400	IP	14 weeks	Extract of Fructus Cannabis (EFC); 200, 400 mg/kg; 14 weeks; Ig	Cardiac Index↓——↑	EFC mitigated the features of ageing induced by D‐gal in rats and relieved age‐related memory impairments
JCR Q4 IF:2.0^28^	Wistar rats	180–220 g	150	——	8 weeks	Mangiferin; 50 mg/kg/day, 100 mg/kg/day; 8 weeks; Ig	Heart index (HI)↑——↓;	Mangiferin suppressed D‐gal‐induced cardiac ageing, and ameliorated cardiac oxidative stress, inflammation, and fibrosis possibly via inhibiting TGF‐β/p38/MK2 signalling pathway
JCR Q3 IF:3.6^29^	Kunming mice	4 weeks	500	SC	4 weeks	*Lactobacillus plantarum* NJAU‐01; 10^7^, 10^8^, and 10^9^ CFU/ml; 4 weeks; Ig	Heart index↑	*L. plantarum* NJAU‐01 alleviated the oxidative damage induced by D‐galactose to the body
JCR Q3 IF:3.6^30^	Wistar rats	170–220 g	150	IP	8 weeks	Resveratrol, 1 mg/kg/day, 8 weeks, Ig; calcitriol, 0.1 μg/kg/day, 8 weeks, IP; resveratrol + calcitriol, 8 weeks	Cardiac klotho↔HW/BW↑——↓	Co‐administration of resveratrol and vitamin D protected the heart against ageing‐induced damage by the modulation of hemodynamic parameters and antioxidant status of the heart
JCR Q2 IF:6.3^32^	C57BL/6	6 weeks	150	IP	10 weeks	Camphorquinone (CQ); 5 mg/kg/day; 8 weeks (starting from the 3rd week of D‐Gal injection); IP	P53, P21↑——↓	CQ possessed antisenescence and cardioprotective properties, and that oxidative‐stress‐induced senescence could be suppressed by AMPK/SIRT1 and autophagy mechanisms
JCR Q2 IF:5.3^33^	C57BL/6J	8 weeks	200	SC	8 weeks	Alginate oligosaccharide(AOS); 50, 100, 150 mg/kg/day; 4 weeks (The last four weeks of the D‐gal injection); Ig	p53, p21↑——↓	AOS alleviated D‐galactose‐induced cardiac ageing via regulating myocardial mitochondria function and integrity in mice
JCR Q3 IF:5.1^38^	C57BL/6J	—	150	IP	8 weeks	CDDO‐imidazolide (CDDO‐Im); 3 μmol/kg/day; 8 weeks; IP	β‐gal, P21↑	Nrf2 activator CDDO‐Im effectively protected against D‐galactose‐induced cardiac ageing by inhibiting oxidative stress in Nrf2+/+ mice (wild‐type mice)
JCR Q2 IF:6.5^40^	C57BL/6	8 weeks	50	SC	8 weeks	NaHS; 10, 50, 100 μmol/kg/day; 8 weeks; IP	p16 ↔, p53 ↑——↓(50↔), p21↑——↓(100↔)	NaHS treatment protected against D‐gal‐accelerated ageing by reducing oxidative stress and increasing eNOS expression and NO contents as well as increasing endogenous H2S production
JCR Q2 IF:5.9^51^	Wistar rats	120 *±* 20 g	200	SC	42 days	Thymoquinone (20 mg/kg, oral); Curcumin (20 mg/kg, oral); Thymoquinone + Curcumin (20 mg/kg + 20 mg/kg); 42 days	TP53, P21↑——↓	D‐gal induced histopathological changes in the heart, besides significantly enhancing apoptosis. TQ and Cur defeated the oxidative alterations of the heart activated by D‐gal. The TQ and Cur combination exhibited more protection for brain and heart tissues than TQ or Cur supplemented alone
JCR Q2 IF:5.9^53^	C57BL/6	6 weeks	150	IP	10 weeks	Licochalcone D (LicoD); 0.5 mg/kg/day; 8 weeks (from the third week of the D‐gal injection); IP	P53, P21↑——↓	This drug had antioxidant, anti‐ageing, and cardioprotective effects, and the activation of AMPK and autophagy ameliorated oxidative stress‐induced senescenc
JCR Q4 IF:2.7^54^	Kunming mice	8 weeks	200	SC	30 days	17β‐Estradiol; 0.016 mg/kg/four days; 30 days; SC	pRb/Rb↑——↓	17β‐E2 downregulated DNA methylation of the Beclin1, LC3, and Atg5 genes, thereby promoting autophagy and delaying cardiac ageing

Abbreviations: Ig, intragastric administration; IP, intraperitoneal; SC, subcutaneous; ↑, indicators increased under the action of D‐galactose; ↑——↓, indicators increased under the action of galactose and decreased under the intervention; ↓——↑, indicators decreased under the action of D‐galactose and increased under the intervention; ↔, there was no change in the indicators under D‐galactose or intervention; (↔), under the intervention treatment of this dose, the indicators did not reverse the change caused by D‐galactose; in addition to the special notes in brackets, the intervention works together with D‐galactose.

The cardiac index is an indicator of cardiac ageing. Interestingly, different changes in the cardiac index were observed under different experimental conditions. Intraperitoneal injection of D‐galactose at a dose of 120 mg/kg/day for six weeks induces oxidative stress and ageing, causes tissue atrophy and finally leads to a decline in the cardiac index in mice.^22^, ^23^, ^24^, ^25^, ^26^ Chen et al.^27^ found that intraperitoneal injection of D‐galactose at a dose of 400 mg/kg/day for 14 weeks decreased the cardiac index. However, some studies have reported the opposite result. Intraperitoneal injection of D‐galactose at a dose of 150 mg/kg/day in Wistar rats for eight weeks, or subcutaneous injection of D‐galactose at a dose of 500 mg/kg/day in mice for four weeks increased the cardiac index and led to cardiac fibrosis.^28^, ^29^, ^30^ This difference may be related to the dose and duration of D‐galactose treatment. In conclusion, taking rats as an example, intraperitoneal injection of a higher dose of D‐galactose and for a prolonged period leads to a decrease in the cardiac index.^27^, ^30^ All discoveries are summarized in Table 1.

## 3 EFFECT OF D‐GALACTOSE ADMINISTRATION ON CARDIAC OXIDATIVE STRESS AND INFLAMMATION

Previous studies have shown that D‐galactose can aggravate cardiac oxidative stress (Table 2). According to Harman D, the ageing process involves the attack of free radicals (usually produced during cell metabolism) on cellular components.^31^ ROS are common free radicals. Excessive D‐galactose administration contributes to increased ROS formation, which subsequently leads to oxidative stress and cardiomyocyte damage. Several studies have assessed the suitability of D‐galactose administration at doses of 50 and 500 mg/kg/day for 6–10 weeks for the establishment of a D‐galactose‐induced cardiac ageing model and reported a close relationship between the initial age of the model animal, dose and duration of treatment. There exists a negative correlation between the age of mice and rats and treatment duration at a dose of 150 or 200 mg/kg/day D‐galactose.^13^, ^32^, ^33^, ^34^, ^35^, ^36^ Moreover, studies have shown that heavier Wistar rats require higher amounts of D‐galactose at the same administration time.^10^, ^12^, ^28^, ^30^, ^37^

**TABLE 2:** Effect of D‐galactose administration on cardiac oxidative stress and inflammation

Ref	Study model	Age	Dose (mg/kg/day)	Route	Duration	Intervention	Major findings	Interpretation
JCR Q2 IF:7.7^10^	Wistar rats	200–220 g	150	SC	4 or 8 weeks	—	MDA↑; TNF‐α↑	D‐galactose‐induced ageing aggravated cardiac oxidative status in obese insulin‐resistant rats
JCR Q3 IF:3.4^11^	Kunming mice	6–8 weeks	500	SC	60 days	PSP; 200, 400 mg/kg/day; 60 days; Ig	ROS, MDA↑——↓; SOD↓——↑	PSP attenuated D‐gal‐induced cardiac ageing via inhibiting oxidative stress
JCR Q3 IF:5.7^12^	Wistar rats	200–220 g	150	SC	8 weeks (after 12 weeks to induce obese‐insulin‐resistant condition by eating a high‐fat diet)	HBOT; 100% oxygen (O_2_) with 250 L/min flow rate; 80 min; once daily for 14 days (after 8 weeks d‐gal injection)	MDA↑——↓; TNF‐α↑——↓	HBOT effectively alleviated cardiac dysfunction via attenuating mitochondrial dysfunction in pre‐diabetic rats
JCR Q4 IF:2.6^13^	SD rats	8 weeks	150	——	8 weeks	AOF; 50, 100, 150 mg/kg/day; 10 weeks; orally	HO1 and Cu/ZN SOD↓——↑(50↔)	AOF negatively modulated the D‐galactose‐induced cardiac hypertrophy signalling mechanism to attenuate ventricular hypertrophy
JCR Q3 IF:2.9^22^	ICR mice	10 weeks	120	IP	6 weeks	ITPL; 50 mg/kg, 100 mg/kg; 10 weeks (pretreated 4 weeks and then treated with D‐galactose for 6 weeks); Ig	MDA, NO↑——↓; SOD, GSH, GSH‐Px↓——↑	ITPL increased superoxide dismutase, glutathione peroxidase, and glutathione levels and reduced nitric oxide and malondialdehyde levels in the serum in oxidative damaged mice induced by D‐gal
JCR Q1 IF:4^24^	Kunming mice	10 weeks	120	IP	6 weeks	*Lactobacillus plantarum* KSFY02; 1.0 × 10^9^ CFU/kg; 10 weeks(pretreat 4 weeks and then 6 weeks during the D‐galactose injections); Ig	MDA, NO↑——↓; SOD, GSH, G SH‐Px↓——↑	LP‐KSFY02 effectively inhibited the decrease in organ indices caused by oxidative ageing and alleviated body tissue atrophy caused by D‐galactose
JCR Q4 IF:2.0^28^	Wistar rats	180–220 g	150	——	8 weeks	Mangiferin; 50 mg/kg/day, 100 mg/kg/day; 8 weeks; Ig	MDA↑——↓; SOD, C AT↓——↑; IL‐1β, IL‐6, T NF‐α↑——↓	Mangiferin suppressed D‐gal‐induced cardiac ageing, ameliorated cardiac oxidative stress, inflammation and fibrosis possibly via inhibiting TGF‐β/p38/MK2 signalling pathway
JCR Q3 IF:3.6^29^	Kunming mice	4 weeks	500	SC	4 weeks	*Lactobacillus plantarum* NJAU‐01; 10^7^, 10^8^, and 10^9^ CFU/ml; 4 weeks; Ig	MDA↑——↓; T‐AOC, SOD, GSH‐PX, CAT↓——↑	*L. plantarum* NJAU‐01 alleviated the oxidative damage induced by D‐galactose to the body and the strain concentration are related to the antioxidant effect
JCR Q3 IF:3.6^30^	Wistar rats	170–220 g	150	IP	8 weeks	Resveratrol, 1 mg/kg/day, 8 weeks, Ig; Calcitriol, 0.1 μg/kg/day, 8 weeks, IP; Resveratrol + calcitriol; 8 weeks	MDA↑——↓; Cu/ZN SOD, Mn‐SOD, CAT mRNA, A and CAT activity ↓——↑; SOD↔——↑	Co‐administration of resveratrol and vitamin D protected the heart against ageing‐induced damage by the modulation of hemodynamic parameters and antioxidant status of the heart
JCR Q3 IF:2.4^34^	Kunming mice	7–8 weeks	200	SC	6 weeks	Pine nut protein hydrolysate (PNPH); 150 mg/kg, 300 mg/kg, and 1000 mg/kg; 6 weeks; Ig	MDA↑——↓, SOD↓——↑, GSH‐Px↓——↑(150↔)	PNPH had antioxidant and anti‐ageing activities in vivo. It could reduce the oxidative damage in heart of mice and inhibit lipid peroxidation, thereby delaying the ageing process of mice induced by D‐galactose
JCR Q3 IF:6.9^35^	C57BL/6J	6 weeks	200	SC	10 weeks	4% H2 inhalation; 4% (v/v) H2 gas for 2 h; 10 weeks	MDA, LPO↑——↓	H2 prevented oxidative stress in D‐galactose‐induced ageing mice when administered by different routes
						H2‐rich water drinking; concentration of H2 is above 600 μmol/L and could be drunk freely; 10 weeks	LPO↑——↓	
						H2‐rich saline injection; 0.1 ml/10 g bw/day; 10 weeks; IP	—	
JCR Q2 IF:6.3^32^	C57BL/6	6 weeks	150	IP	10 weeks	CQ; 5 mg/kg/day; 8 weeks (starting from the 3rd week of D‐Gal injection); IP	IL‐1α, IL‐1β, IL‐6↑——↓	CQ possessed antisenescence and cardioprotective properties, and that oxidative‐stress‐induced senescence was suppressed by AMPK/SIRT1 and autophagy mechanisms
JCR Q2 IF:5.3^33^	C57BL/6J	8 weeks	200	SC	8 weeks	AOS; 50, 100, 150 mg/kg/day; 4 weeks (The last four weeks of the D‐gal injection); Ig	ROS, MDA↑——↓; p47‐phox, p67‐phox and gp91‐phox↑——↓	AOS alleviated D‐gal‐induced cardiac ageing via regulating myocardial mitochondria function and integrity in mice
JCR Q2 IF:4.1^36^	Wistar rats	18 weeks	150	—	4 weeks	AOF; 100 mg/kg/day; orally administered ADMSCs; administered intravenously with ADMSCs of 10^7^ cells	Rac‐1, Nox‐2↑——↓; HO‐1 and Cu/ZN SOD↓——↑ IkB↓——↑; p‐NF‐κB, p65, IL‐6↑——↓	Synergistic effects of AOF and ADMSCs together possessed therapeutic values against cardiac ageing induced by D‐gal
JCR Q2 IF:5.1^37^	Wistarrats	130–150 g	200	IP	8 weeks	Zeaxanthin heneicosylate(ZH); 250 μg/kg; 4 weeks after 8 weeks d‐gal injection; orally	SOD↓—↑, iNOS↑——↓; IL‐6, NF‐κB↑——↓	ZH isolated from *D. salina* ameliorated age‐associated cardiac dysfunction in rats through the activation of retinoid receptors
JCR Q3 IF:5.1^38^	C57BL/6J	—	150	IP	8 weeks	CDDO‐Im; 3 μmol/kg/day; 8 weeks; IP	MDA, NO, and PC↑——↓; CAT, SOD, GSH‐Px↓——↑; HO‐1, SOD‐1↓	Nrf2 activator CDDO‐Im effectively protected against D‐galactose‐induced cardiac ageing by inhibiting oxidative stress in Nrf2+/+ mice (wild‐type mice)
JCR Q2 IF: 6.6^39^	ICR mice	6 weeks	120	IP	6 weeks	Antarctic Ice Microalgae Polysaccharides (AIMP); 50 mg/kg or 100 mg/kg; 6 weeks; Ig	MDA, NO↑——↓; SOD, GSH, GSH‐Px↓——↑	AIMP effectively inhibited oxidative damage in mice with D‐galactose‐induced oxidative damage
JCR Q2 IF:6.5^40^	C57BL/6	8 weeks	50	SC	8 weeks	NaHS; 10, 50, 100 μmol/kg/day; 8 weeks; IP	ROS↑——↓; SOD, GPx, NO↓——↑; CSE↓——↑(10↔); CBS↔ ——↑(10↔)and 3‐MST↔ H2S↓——↑(10, 50↔)	NaHS treatment protected against D‐gal‐accelerated ageing by reducing oxidative stress and increasing eNOS expression and NO contents as well as increasing endogenous H2S production
JCR Q3 IF:1.9^71^	Kunming mice	18–22 g	125	SC	10 weeks	*Dendrobium officinale* (DO); DO‐1 (DO juice with a dose of 1 g/kg), DO‐2 (DO Polysaccharide with a dose of 0.32 g/kg); 9 weeks(from ten days after injection of D‐gal); orally	NO↔, SOD↓——↑	DO had a marked anti‐ageing effect on the D‐galactose‐induced model of ageing

Abbreviation: Ig, intragastric administration; IP, intraperitoneal; SC, subcutaneous; ↑, indicators increased under the action of D‐galactose; ↓, indicators decreased under the action of D‐galactose; ↑——↓, indicators increased under the action of galactose and decreased under the intervention; ↓——↑, indicators decreased under the action of D‐galactose and increased under the intervention; ↔, there was no change in the indicators under D‐galactose or intervention; (↔), under the intervention treatment of this dose, the indicators did not reverse the change caused by D‐galactose; ↔——↑, the indicators did not change after D‐galactose administration, but increased after intervention; in addition to the special notes in brackets, the intervention works together with D‐galactose.

Evidence suggests that D‐galactose treatment increases oxidants levels and decreases antioxidants levels (Table 2). ROS can attack proteins, lipids and DNA, altering their structures and functions. Many studies have reported that under D‐galactose treatment, the levels of ROS, malondialdehyde (MDA), lactoperoxidase (LPO), and NADPH oxidase 2 (NOX2) are significantly increased, as described in Table 2. Rac‐1 and protein carbonyl (PC) level also increased after D‐galactose injection.^36^, ^38^ Nrf2, a major stress‐response transcription factor, regulates the expression of heme oxygenase‐1 (HO‐1), Cu/ZN superoxide dismutase (SOD), Nox‐2, Rac‐1 and significantly reduces ageing‐induced oxidative stress in D‐galactose‐induced ageing rat hearts.^36^ The activation of nitric oxide synthases (NOS) and nitric oxide (NO) production triggers the production of O_2_ and OH‐free radicals, leading to cell damage.^39^ Previous studies have shown that D‐galactose treatment significantly increases the levels of NO and iNOS.^22^, ^37^, ^38^, ^39^, ^40^

The antioxidant enzyme system is an antioxidant system and includes SOD, catalase (CAT), glutathione peroxidase (GSH‐Px) and among others. The total antioxidant capacity (T‐AOC) level of mice significantly decreased after subcutaneous administration of 500 mg/kg/day D‐galactose for 4 weeks.^29^ As summarized in Table 2, SOD, Cu/ZN SOD, and Mn‐SOD levels, the mRNA expression and activity of CAT, and GSH‐PX levels, which represent antioxidant capacity, significantly decreased following D‐galactose administration. In addition, D‐galactose‐induced cardiac ageing models also show reduced HO‐1 and cystathionine gamma lyase (CSE).^36^, ^38^, ^40^ The other antioxidant system is the no‐enzyme antioxidant system, which includes glutathione (GSH), vitamin C, vitamin E and some tracer elements such as copper and zinc. D‐galactose administration at a dose of 120 mg/kg/day for six weeks has been shown to induce GSH reduction in ICR mice.^22^, ^39^

Oxidative stress plays a vital role in the activation of transcription factors‐mediated signalling pathways, such as the nuclear factor kappa B (NF‐κB) pathway, which can lead to ageing by regulating the expression of inflammatory genes.^41^ D‐galactose through the activation of NF‐κB inflammatory signalling pathway promotes the release of inflammatory factors, accelerating the formation of an inflammatory state and ageing.^36^ NF‐κB, a transcription factor, is inhibited and inactivated by inhibitory κB (IκBs) proteins.^42^ IκBα inhibits NF‐κB function by binding. Under ROS excess, IκBα is phosphorylated and degraded to form p‐IκBα, and unbound NF‐κB enters the nucleus and activates inflammatory responses.^43^, ^44^ Studies have shown that p‐NF‐κB, NF‐κB, P65 (a member of the NF‐κB family of proteins), IL‐1α, IL‐1β, and IL‐6 increase, and IkB decreases with D‐galactose administration.^12^, ^28^, ^32^, ^36^, ^37^

## 4 EFFECT OF D‐GALACTOSE ADMINISTRATION ON CARDIAC APOPTOSIS AND AUTOPHAGY

As summarized in Table 3, D‐galactose administration aggravates apoptosis and decreases cardiac autophagy. D‐galactose intraperitoneal or subcutaneous injection, at a dose of 150–200 mg/kg/day for 4 and 8 weeks alters apoptosis‐related indices. D‐galactose increased the number of cardiac apoptosis cells.^5^, ^10^, ^12^, ^38^ D‐galactose also activates apoptosis pathways, including the mitochondria‐initiated intrinsic pathway and death receptor‐stimulated extrinsic pathway, involved in cardiomyocytes apoptosis.^45^ The extrinsic apoptotic pathway is usually triggered by the Fas ligand (FAS‐L), which induces receptor activation and leads to the formation of a death‐inducing signalling complex (DISC). DISC recruits and activates pro‐caspase 8, and then activates the downstream effector caspase‐3, which can directly degrade structural and functional proteins and cause apoptosis.^46^, ^47^ A study reported that FAS‐L and caspase‐8 levels increased after D‐galactose injection, corroborating the role of D‐galactose in apopttosis.^5^ The intrinsic pathway (also known as the mitochondrial pathway) involves many signalling proteins. D‐galactose also induces cardiac cell apoptosis, which is strictly regulated by pro‐apoptotic and anti‐apoptotic factors of Bcl‐2 family. The pro‐apoptotic factor Bax forms holes in the outer mitochondrial membrane, destroying the mitochondrial membrane integrity. This leads to increased mitochondrial cytochrome c release and activation of caspase 3.^48^, ^49^, ^50^ Many studies have shown that D‐galactose‐induced cardiac cell apoptosis is characterized by an increase in Bax and cleaved caspase‐3, and a decrease in the gene and protein expression of anti‐apoptotic factor Blc2.^5^, ^10^, ^12^, ^50^, ^51^

**TABLE 3:** Effect of D‐galactose administration on cardiac autophagy and apoptosis

Ref	Study model	Age	Dose (mg/kg/day)	Route	Duration	Intervention	Major findings	Interpretation
JCR Q2 IF:10.5^5^	SD‐rats	4 weeks	150	IP	8 weeks	Exercise training; In the first two weeks, swimming for 20 min/day, 5 times/week. The duration of swimming was extended to 30 min/−day starting from the 3rd week and to 60/min during the fourth to eighth weeks; 8 weeks	Apoptosis:number of apoptotic cells in the LV↑——↓; Extrinsic pathway: Fas‐L(↑——↔), FADD↔, and caspase‐8↑——↓; Intrinsic pathway: Bax and cleaved caspase‐3(↑——↔), PARP↑——↓	Exercise training attenuated ageing‐associated cardiac apoptosis and cardiac fibrosis induced by D‐galactose
JCR Q2 IF:7.7^10^	Wistar rats	200–220 g	150	SC	4 weeks or 8 weeks	——	Apoptosis: Cleaved caspase‐3/caspase‐3↑, TUNEL‐positive cells↑ Autophagy impairment: Beclin‐1↓(4 weeks↔), P62	D‐galactose‐induced ageing led to a worsening of cardiac cells apoptosis in obese insulin‐resistant rats and exacerbated the impairment of autophagic processes in obese insulin‐resistant rats
JCR Q3 IF:5.7^12^	Wistar rats	200–220 g	150	SC	8 weeks (after 12 weeks to induce obese‐insulin resistant condition by eating a high‐fat diet)	HBOT; 100% oxygen (O_2_) with 250 L/min flow rate; 80 min; once daily for 14 days (after 8 weeks d‐gal injection)	Apoptosis: TUNEL assay, Bax/Bcl‐2 ratio, and cleaved‐caspase 3/caspase 3↑——↓ Autophagy: Beclin‐1↓——↑, p62 ↑——↓, LC3II↔	HBOT effectively alleviated cardiac dysfunction via attenuating mitochondrial dysfunction in pre‐diabetic rats
JCR Q2 IF:6.3^32^	C57BL/6	6 weeks	150	IP	10 weeks	CQ; 5 mg/kg/day; 8 weeks (starting from the 3rd week of D‐Gal injection); IP	Autophagy: LC3II/LC3Iand BECN1↓——↑; SQSTM1↑——↓	CQ possessed antisenescence and cardioprotective properties, and that oxidative‐stress‐induced senescence was suppressed by AMPK/SIRT1 and autophagy mechanisms
JCR Q2 IF:5.3^33^	C57BL/6J	8 weeks	200	SC	8 weeks	AOS; 50, 100, 150 mg/kg/day; 4 weeks (The last four weeks of the D‐gal injection); Ig	Autophagy: beclin‐1↓——↑; mTOR phosphorylation↑——↓	AOS alleviated D‐gal‐induced cardiac ageing via regulating myocardial mitochondria function and integrity in mice
JCR Q3 IF:5.1^38^	C57BL/6J	—	150	IP	8 weeks	CDDO‐Im; 3 μmol/kg/day; 8 weeks; IP	TUNEL positive↑	Nrf2 activator CDDO‐Im effectively protected against D‐galactose‐induced cardiac ageing by inhibiting oxidative stress in Nrf2+/+ mice (wild‐type mice)
JCR Q2 IF:5.9^51^	Wistar rats	120 *±* 20 g	200	SC	42 days	Thymoquinone (20 mg/kg, oral); Curcumin (20 mg/kg, oral); Thymoquinone + Curcumin (20 mg/kg + 20 mg/kg); 42 days	Apoptosis: cardiac necrosis↑——↓; Necrosis and apoptosis: Casp‐3 and Bax genes, caspase 3 protein↑——↓; Blc2 gene and protein↓——↑	D‐gal induced histopathological changes in the heart, besides significantly enhancing apoptosis. TQ and Cur defeated the oxidative alterations of the heart activated by D‐gal. The TQ and Cur combination exhibited more protection for brain and heart tissues than TQ or Cur supplemented alone
JCR Q2 IF:5.9^53^	C57BL/6	6 weeks	150	IP	10 weeks	Lico D; 0.5 mg/kg/day; 8 weeks (From the third week of the D‐gal injection); IP	Autophagy: LC3II, BECN1↓——↑; SQSTM1↑——↓	This drug had antioxidant, anti‐ageing, and cardioprotective effects, and the activation of AMPK and autophagy ameliorated oxidative stress‐induced senescence
JCR Q4 IF:2.7^54^	Kunming mice	8 weeks	200	SC	30 days	17β‐Estradiol; 0.016 mg/kg/four days; 30 days; SC	Autophagy: the expression of Beclin1, LC3, and Atg5 gene and protein↓——↑; P62↑——↓; Beclin1, LC3, and Atg5 methylation levels↑——↓	17β‐E2 downregulated DNA methylation of the Beclin1, LC3, and Atg5 genes, thereby promoting autophagy and delaying cardiac ageing

Abbreviations: Bax, Bcl‐2‐associated X protein; Bcl‐2, B‐cell lymphoma 2; Cyt‐c, cytochrome c; FADD, Fas‐associated death domain; Fas, tumour necrosis factor receptor; Ig, intragastric administration; IP, intraperitoneal; mito, mitochondria; SC, subcutaneous; ↑, indicators increased under the action of D‐galactose; ↓, indicators decreased under the action of D‐galactose; ↑——↓, indicators increased under the action of galactose and decreased under the intervention; ↓——↑, indicators decreased under the action of D‐galactose and increased under the intervention; ↔, there was no change in the indicators under D‐galactose or intervention;(↔), under the intervention treatment of this dose, the indicators did not reverse the change caused by D‐galactose; ↑——↔, the indicators increased after D‐galactose administration, but did not change after intervention; in addition to the special notes in brackets, the intervention works together with D‐galactose.

Autophagy, an intracellular catabolic recycling system associated with life and health span extension, is a fundamental process that maintains cardiac and vascular health during ageing.^52^ The subcutaneous or intraperitoneal administration of D‐galactose at a dose of 150–200 mg/kg/day for 4–10 weeks, reduced autophagy and accelerated cardiac ageing (Table 3). Several factors regulate the occurrence and development of autophagy. Lower levels of lipidated microtubule‐associated protein light chain 3 (LC3‐II), a marker of autophagosome formation, have been observed in D‐galactose‐induced ageing hearts.^32^, ^53^, ^54^ Beclin1, an essential autophagy adjustment factor, is also reduced in the ageing heart.^10^, ^12^, ^33^, ^54^ Beclin1 abnormality in the promoter region is related to the methylation status, and abnormal methylation of autophagy genes can regulate autophagy, inducing the onset and development of cardiac ageing.^54^, ^55^ Moreover, BECN1 and Atg5 levels significantly decreased, while SQSTM1, P62 and mTOR phosphorylation increased (Table 3). Interestingly, D‐galactose‐induced ageing aggravates autophagy impairment in the cardiac tissues in a time‐dependent manner.^10^ Thus, D‐galactose impairs cardiac autophagy, which is essential for maintaining cardiac health during ageing.

## 5 EFFECT OF D‐GALACTOSE ADMINISTRATION ON CARDIAC MITOCHONDRIA

D‐galactose administration reduces cardiac mitochondrial function. Previous studies have shown that mitochondrial integrity decreases with ageing, demonstrating the importance of mitochondrial dysfunction in cell senescence.^56^ In addition to the loss of mitochondrial integrity, age‐related mitochondrial dysfunction is characterized by increased mitochondrial reactive oxygen species (MitoROS) formation, decreased mitochondrial membrane potential (MMP), and mitochondrial biogenesis efficiency, alterations in mitochondrial dynamics, and defective quality control by autophagy.^57^ As described in Table 4, subcutaneously injection of D‐galactose at a dose of 125–200 mg/kg/day for 6–10 weeks significantly negatively altered cardiac mitochondrial functions. Some studies have shown that D‐galactose administration significantly increases MitoROS in the heart.^10^, ^12^ Additionally, D‐galactose also leads to enlarged cardiomyocyte mitochondria with swelling and partial loss of cristae, decreased MMP and increased depolarization of mitochondrial membranes.^10^, ^12^, ^33^ The study demonstrates that mitochondrial biogenesis decreases in the aged hearts, as indicated by the decreases in mtDNA copy number and peroxisome proliferator‐activated receptor‐γ coactivator‐1α(PGC‐1α).^33^ SIRT3 also plays a vital role in maintaining mitochondrial bioenergetics. SIRT3 protein expression significantly decreased in D‐galactose‐induced ageing mice.^33^ D‐galactose administration at a dose of 150 mg/kg/day, for eight weeks significantly reduced the cardiac mitochondrial fusion marker mitofusin 1 and 2 (MFN1 and MFN2) and increased the levels of dynamin‐related protein 1(Drp1) and pDrp1^ser616^, indicating mitochondrial dynamics imbalance.^10^, ^12^ Additionally, impaired mitophagy is associated with an accelerated decline in mitochondrial integrity and heart function.^52^ Studies have shown that D‐galactose administration decreased PINK1, Parkin, and Sirt6.^21^

**TABLE 4:** Effect of D‐galactose administration on the cardiac mitochondria

Ref	Study model	Age	Dose (mg/kg/day)	Route	Duration	Intervention	Major findings	Interpretation
JCR Q2 IF:7.7^10^	Wistar rats	200–220 g	150	SC	4 weeks or 8 weeks	—	Mitochondrial impairment↑; mitochondrial ROS↑; depolarization of mitochondrial membranes and Cardiac mitochondrial swelling↑; MFN1, MFN2↓; pDrp1^ser616/^/Total Drp1↑, Drp1/VDAC↑	D‐galactose‐induced ageing exacerbated cardiac mitochondrial dysfunction in obese insulin‐resistant rats, and both D‐galactose‐induced ageing and obese insulin resistance led to the impairment of the cardiac mitochondrial fusion process
JCR Q3 IF:5.7^12^	Wistar rats	200–220 g	150	SC	8 weeks (after 12 weeks to induce obese‐insulin resistant condition by eating a high‐fat diet)	HBOT; 100% oxygen (O_2_) with 250 L/min flow rate; 80 minutes; once daily for 14 days (after 8 weeks d‐gal injection)	Mitochondrial function: mitochondrial ROS level↑——↓; depolarization, and swelling↑——↓Mitochondrial dynamics processes: MFN1 and MFN 2↓——↔	HBOT effectively alleviated cardiac dysfunction via attenuating mitochondrial dysfunction in pre‐diabetic rats
JCR Q3 IF:5.7^21^	C57BL/6	8–10 weeks	150	SC	10 weeks	Natural flavone acacetin; 10, 20, 50 mg/kg/day; 10 weeks; Ig	Mitophagy: PINK1, Parkin, and Sirt6 (10 mg/kg/day↔)↓——↑	Acacetin significantly inhibited in vivo cardiac ageing induced by D‐galactose via Sirt1‐mediated activation of the Sirt6/AMPK signalling pathway, thereby enhancing mitophagy and preserving mitochondrial function
JCR Q2 IF:5.3^33^	C57BL/6J	8 weeks	200	SC	8 weeks	AOS; 50, 100, 150 mg/kg/day; 4 weeks (The last four weeks of the D‐gal injection); Ig	Mitochondria: cardiomyocyte mitochondria were enlarged, swelling and partial loss of cristae↑——↓; MMP↓——↑; PGC‐1α↓——↑; SIRT3↓——↑; mtDNA copy number↓——↑	AOS alleviated D‐gal‐induced cardiac ageing via regulating myocardial mitochondria function and integrity in mice
JCR Q4 IF:3^50^	SD rats	3 months	125	SC	6 weeks	Melatonin; 10 mg/kg/day; 6 weeks; IP	Mitochondria: Bcl‐2↓——↑; Cyt‐c protein in the cytoplasm↑——↓	Melatonin exhibited a protective effect on mitochondrial function in a rat model of accelerated ageing

Abbreviations: Ig, intragastric administration; IP, intraperitoneal; SC, subcutaneous; ↑, indicators increased under the action of D‐galactose; ↓, indicators decreased under the action of D‐galactose; ↑——↓, indicators increased under the action of galactose and decreased under the intervention; ↓——↑, indicators decreased under the action of D‐galactose and increased under the intervention; (↔), under the intervention treatment of this dose, the indicators did not reverse the change caused by D‐galactose; ↓——↔, The indicators decreased after D‐galactose administration, but did not change after intervention; in addition to the special notes in brackets, the intervention works together with D‐galactose.

## 6 EFFECT OF D‐GALACTOSE ADMINISTRATION ON CARDIAC HISTOPATHOLOGY AND FUNCTION

Cardiac remodelling and left ventricular (LV) dysfunction are the primary manifestations of cardiac ageing.^58^, ^59^ Several studies have demonstrated an increase in heart weight, cardiac hypertrophy, cardiac fibrosis, and cardiac remodelling in D‐galactose‐induced cardiac ageing models (Table 5). D‐galactose administration of 120–500 mg/kg/day for 4–8 weeks has been reported to cause an increase in the whole heart weight (WHW), left ventricular weight (LVW), and LV wall thickening, an increase in hypertrophic makers such as ANP, BNP, β‐MHC, and MYH7 and a decrease in MYH6.^13^, ^28^, ^33^, ^36^ Chang et al.^13^ found that the concentric hypertrophy‐related MAPKs such as p‐ERK1/2, p‐c‐JUN, p‐JNK, and p‐p38, pathological hypertrophy‐associated transcription factors such as NFATc3 and p‐GATA4, and the eccentric pathological related protein p‐MEK5, p‐ERK5, and transcription factors STAT3 were significantly increased with D‐galactose administration.

**TABLE 5:** Effect of D‐galactose administration on the cardiac morphology and function

Ref	Study model	Age	Dose (mg/kg/day)	Route	Duration	Intervention	Major findings	Interpretation
JCR Q2 IF:10.5^5^	SD‐rats	4 weeks	150	IP	8 weeks	Exercise training; In the first two weeks, swimming for 20 min/day, 5 times/week. The duration of swimming was extended to 30 min/−day starting from the 3rd week and to 60/min during the fourth to eighth weeks; 8 weeks	Cardiac fibrosis and collagen accumulation↑——↓	Exercise training attenuated ageing‐associated cardiac apoptosis and cardiac fibrosis induced by D‐galactose
JCR Q2 IF:6.2^9^	C57BL/6	3 months	120	SC	8 weeks	Kanglexin(10 and 20 mg/kg/day); Emodin (20 mg/kg); 8 weeks; Ig	Collagen deposition and fibrosis↑——↓; Cardiac diastolic function↑——↓, E/V↓——↑, LV mass↑——↓, EF, FS ↔	Diastolic dysfunction and cardiac remodelling in mice with D‐gal‐induced ageing were markedly mitigated by KLX and emodin
JCR Q2 IF:7.7^10^	Wistar rats	200–220 g	150	SC	4 weeks or 8 weeks	——	LV dysfunction: FS↓; LVESP, dP/dt max and SV↓; LVEDP and dp/dt min↑ Sympathovagal imbalance: The LF/HF ratio of HRV↑	D‐galactose‐induced ageing aggravated LV dysfunction and sympathovagal imbalance in obese insulin‐resistant rats
JCR Q3 IF:3.4^11^	Kunming mice	6–8 weeks	500	SC	60 days	PSP; 200, 400 mg/kg/day; 60 days; Ig	Disorder of cardiac fibre arrangement↑——↓; CK and cTnT↑——↓	PSP attenuated D‐gal‐induced cardiac ageing via inhibiting oxidative stress
JCR Q3 IF:5.7^12^	Wistar rats	200–220 g	150	SC	8 weeks (after 12 weeks to induce obese‐insulin resistant condition by eating a high‐fat diet)	HBOT; 100% oxygen (O_2_) with 250 L/min flow rate; 80 min; once daily for 14 days (after 8 weeks d‐gal injection)	LVEF, FS, LVESP, dP/dt max, SV↓——↑; LVEDP, dP/dt min↑——↓, HR↔; LF/HF↑——↓	HBOT effectively alleviated cardiac dysfunction via attenuating mitochondrial dysfunction in pre‐diabetic rats
JCR Q4 IF:2.6^13^	SD rats	8 weeks	150	——	8 weeks	AOF; 50, 100, 150 mg/kg/day; 10 weeks; orally	WHW and LVW↑——↓(50↔); LV wall thickening↑——↓ Cardiac Hypertrophy: p‐ERK1/2, p‐JNK, p‐p38↑——↓(50↔); p‐c‐JUN↑——↓; NFATc3 and p‐GATA4↑——↓(50, 100↔); p‐ERK5↑——↓, p‐MEK5, STAT3↑——↓(50, 100↔); MYH6↓——↑, MYH7↑——↓BNP↑——↓(50↔); LVPWd↑——↓(50↔)	AOF negatively modulated the D‐galactose‐induced cardiac hypertrophy signalling mechanism to attenuate ventricular hypertrophy
JCR Q3 IF:5.7^21^	C57BL/6	8–10 weeks	150	SC	10 weeks	Natural flavone acacetin; 10, 20, 50 mg/kg/day; 10 weeks; Ig	EF, FS↓——↑; LVAWd, LVPWd↓——↑(10 mg/kg/day↔); LVESD↔	Acacetin significantly inhibited in vivo cardiac ageing induced by D‐galactose via Sirt1‐mediated activation of Sirt6/AMPK signalling pathway, thereby enhancing mitophagy and preserving mitochondrial function
JCR Q4 IF:2.0^28^	Wistar rats	180–220 g	150	——	8 weeks	Mangiferin; 50 mg/kg/day, 100 mg/kg/day; 8 weeks; Ig	Cardiac morphology: heart weight ↑——↓; hypertrophic makers ANP, BNP↑——↓, β‐MHC↑——↓(50↔); Cardiac collagen deposition: Masson‐positive and Sirius red‐positive area↑——↓; pro‐fibrogenic proteins TGF‐β, p‐p38/p38, p‐MK2/MK2↑——↓; Col‐I, Col‐III, and α‐SMA↑——↓; Cardiac function: CK and LDH in serum↑——↓	Mangiferin suppressed D‐gal‐induced cardiac ageing, ameliorated cardiac oxidative stress, inflammation and fibrosis possibly via inhibiting TGF‐β/p38/MK2 signalling pathway
JCR Q3 IF:3.6^30^	Wistar rats	170–220 g	150	IP	8 weeks	Resveratrol, 1 mg/kg/day, gavage, 8 weeks; Calcitriol, 0.1 μg/kg/day, IP, 8 weeks; resveratrol + calcitriol; 8 weeks	Cardiomyocytes size and cardiac fibrosis↑——↓	Co‐administration of resveratrol and vitamin D protected the heart against ageing‐induced damage by the modulation of hemodynamic parameters and antioxidant status of the heart
JCR Q2 IF:5.3^33^	C57BL/6J	8 weeks	200	SC	8 weeks	AOS; 50, 100, 150 mg/kg/day; 4 weeks (The last four weeks of the D‐gal injection); Ig	Cardia morphology: disordered arrangement of cardiomyocytes and increased intercellular space between cells left ventricular cardiomyocyte area↑——↓; cardiac fibrosis↑——↓ Cardiac function: EF, FS↓——↑; heart rate ↔; LVEDD↔; LVESD↑——↓(low‐dose AOS↔); LVPWd↔; ANP, BNP↑——↓	AOS alleviated D‐gal‐induced cardiac ageing via regulating myocardial mitochondria function and integrity in mice
JCR Q2 IF:4.1^36^	Wistar rats	18 weeks	150	—	4 weeks	AOF; 100 mg/kg/day; orally administered ADMSCs; administered intravenously with ADMSCs of 10^7^ cells	Size and weight of heart left ventricular weight↑——↓; Cardiac hypertrophy: ANP, BNP↑——↓; Cardiac fibrosis: deposition of collagen↑——↓; CTGF, MMP‐2, and MMP‐9↑——↓; TIMP‐1, −4↓——↑	Synergistic effects of AOF and ADMSCs together possessed therapeutic values against cardiac ageing induced by D‐gal
JCR Q2 IF:5.1^37^	Wistar rats	130–150 g	200	IP	8 weeks	ZH; 250 μg/kg; 4 weeks after 8 weeks d‐gal injection; orally	An abnormal myocardial architecture decreased cellular volume with an exhibition of spaces between the cells; heart rate, PR, QRS↑——↓; ECG pattern in ST height and T wave has been improved; homocysteine (HS), creatinine kinase isoenzyme (CK‐MB), lactate dehydrogenase (LDH)in serum↑——↓; Cardiac GLUT‐4 in serum↓——↑	ZH isolated from D. salina ameliorated age‐associated cardiac dysfunction in rats through the activation of retinoid receptors
JCR Q4 IF:2.7^54^	Kunming mice	8 weeks	200	SC	30 days	17β‐Estradiol; 0.016 mg/kg/four days; 30 days; SC	EF and FS↓——↑; LVVd, LV mass, HR↔; LVVs↑——↓	17β‐E2 downregulated DNA methylation of the Beclin1, LC3, and Atg5 genes, thereby promoting autophagy and delaying cardiac ageing
JCR Q2 IF:4.1^60^	SD rats	8 weeks	150	—	8 weeks	AOF; 50, 100, 150 mg/kg/day; 8 weeks; orally	Cardiac fibrosis: MMP 2 and MMP 9↑——↓; cardiac accumulation of collagen fibres↑——↓ (TGFβ1, p‐MEK1/2, p‐ERK1/2, SP1 and CTGF↑——↓); Extracellular collagen degradation: MMP‐9↑——↓	D‐galactose‐induced ageing activated the process of myocardial fibrosis and caused cardiac remodelling. AOF treatment decreased the risk for myocardial fibrosis via down‐regulation of collagen‐related accumulation/degradative pathways and further maintaining collagen homeostasis
JCR Q2 IF:6.5^63^	C57BL/6J	8 weeks	125	SC	3 months	NaHS; 100 μmol/kg/day; 3 months; IP	EF and FS↓——↑	H2S Restored the Diurnal Variations of EF and FS in Subacute Ageing Mice
JCR Q2 IF:5^16^	C57BL/6	10–12 weeks	100	SC	10 weeks	MiR‐21 knockout mice	EF, FS↓——↑	MiR‐21 knockout had a protective effect against D‐gal‐induced cardiac alterations

Abbreviations: Ig, intragastric administration; IP, intraperitoneal; SC, subcutaneous; ↑, indicators increased under the action of D‐galactose; ↓, indicators decreased under the action of D‐galactose; ↑——↓, indicators increased under the action of galactose and decreased under the intervention; ↓——↑, indicators decreased under the action of D‐galactose and increased under the intervention; ↔, there was no change in the indicators under D‐galactose or intervention;(↔), under the intervention treatment of this dose, the indicators did not reverse the change caused by D‐galactose; in addition to the special notes in brackets, the intervention works together with D‐galactose.

As summarized in Table 5, D‐galactose administration increases cardiac fibrosis and collagen deposition.^5^, ^9^, ^28^, ^30^, ^33^, ^36^, ^60^ After activation, excessive collagen secreted by cardiac fibroblasts is deposited in the extracellular matrix, resulting in myocardial fibrosis.^61^ The production of various cytokines and growth factors plays a critical role in the cardiac fibrosis pathway. In the ageing heart, we observed an increase in extracellular matrix (ECM) proteins such as Col‐I, Col‐III, and α‐SMA, as well as pro‐fibrogenic proteins TGF‐β1, p‐p38 and p‐MK2.^28^ Moreover, phosphoMEK1/2 (MAP Kinase Kinase), extracellular signal‐regulated kinases 1/2 (ERK1/2), specific protein 1 (SP1), and connective tissue growth factor (CTGF), all elements of a fibrotic response, increased with D‐galactose administration, resulting in excessive ECM protein production and collagen expression.^36^, ^60^ The homeostatic imbalance of matrix metalloproteinases (MMPs) and tissue inhibitors of metalloproteinases (TIMPs) lead to cardiac fibrosis.^62^ Previous studies have shown that MMP‐2 and MMP‐9 increased and TIMP‐1/4 decreased with D‐galactose administration.^36^, ^60^

D‐galactose‐induced ageing hearts have been shown to show impaired cardiac fibre arrangement, decreased cellular volume with spaces between cells, increased size of cardiomyocytes and area of left ventricular cardiomyocytes.^11^, ^30^, ^33^, ^37^ In summary, tissue regeneration potential is critically affected by the deterioration of the function of stem cells during ageing. D‐galactose‐induced cardiac remodelling can lead to a decline in cardiac function.^36^

As summarized in Table 5, cardiac function was significantly reduced in mice and rats injected with D‐galactose at 100–500 mg/kg/day for 4–12 weeks. Markers of cardiac function include homocysteine (HS), creatinine kinase isoenzyme (CK‐MB), lactate dehydrogenase (LDH), glucose transporter 4 (GLUT‐4), and cardiac troponin T (cTnT) and are measured to assess cardiac function. HS, CK, LDH and cTnT levels in serum increased, and GLUT‐4 levels in serum decreased after D‐galactose injection.^11^, ^28^, ^37^ Echocardiography is the standard method used for evaluating cardiac function. Left ventricular ejection fraction (LVEF) and left ventricular fractional shortening (LVFS) are common test indicators measured to assess cardiac function. D‐galactose has been shown to induce a decrease in LVEF and FS.^12^, ^16^, ^21^, ^33^, ^54^, ^63^ D‐galactose also worsened LV function as indicated by significantly increased LV end‐diastolic pressure (LVEDP), dP/dt min, and LV volume in end systole (LVVs), and significantly reduced LV end‐systolic pressure (LVESP), dP/dt max, and SV.^10^, ^12^, ^54^ Additionally, D‐galactose injection did not significantly alter heart rate,^12^, ^33^, ^54^ and significantly increased LV end‐systolic diameter (LVESD) and LV mass.^9^, ^33^ D‐galactose administration at a dose of 150 mg/kg/day for 10 weeks decreased LV anterior wall thickness (LVAWd) and LV posterior wall thickness (LVPWd) in C57 mice.^21^ However, another study reported that LVPWd increased after D‐galactose administration.^13^ The mitral E/A ratio decreases significantly with the increase in age, indicating impaired ventricular filling.^9^, ^62^ The low‐frequency /high‐frequency (LF/HF) ratio is measured as an indicator of cardiac sympathovagal balance. At a dose of 150 mg/kg/day, D‐galactose administration for eight weeks significantly increased LF/HF in ageing Wistar rats.^10^, ^12^ D‐galactose administration resulted in dramatic changes in the electrocardiographic (ECG) measurements in the form of an irregular rhythm of heartbeats, reduced ST height, increased PR and QRS intervals, and negative T wave.^37^

## 7 SUMMARY OF IN VITRO STUDIES ON THE D‐GALACTOSE‐INDUCED CARDIOMYOCYTES AGEING

Hayflick found the limit of cell division through in vitro cell culture and proposed an advanced hypothesis that the finite lifetime of diploid cells in vitro may be an expression of ageing or senescence at the cellular level.^64^ Cellular senescence is critical in vivo and is associated with age‐related diseases.^15^ Some researchers have used primary cardiomyocytes and H9c2 cell line to study D‐galactose‐induced cardiac ageing. The typical dose of D‐galactose is 10–50 g/L, and the treatment duration is 24–72 h. However, there is one exception, the amount of galactose is tiny, considering that it may be the author's clerical error.^9^ D‐galactose increases ageing markers, oxidative stress and inflammation, apoptosis of cardiomyocytes, and decreases cardiomyocyte autophagy. These effects induced by D‐galactose are concentration and time‐dependent.^65^, ^66^, ^67^, ^68^, ^69^ These results are summarized in Table 6.

**TABLE 6:** Summary of in vitro studies on the D‐galactose‐induced cardiac ageing

Ref	Study model	Dose	Duration	Intervention	Cardiac ageing	Cardiac oxidative stress and inflammation	Apoptosis/autophagy/cardiac mitochondria/cytotoxicity	Interpretation
JCR Q2 IF:6.2^9^	Mice primary cardiomyocytes	40 μM (7.2 mg/L)	24 h	Kanglexin (10 μM and 20 μM), emodin (20 μM)	SA‐β‐gal staining↑——↓; p53, p21↑——↓	ROS↑——↓	Mitochondrial: mitochondrial swelling, myofilament rupture, nuclear constriction, and mitophagy inhibition↑——↓ Mitophagy: P62↑——↓, LC3 II/I, Parkin↓——↑; autolysosome formation↓——↑	KLX and emodin treatment reversed the senescence of neonatal mouse cardiomyocytes and mitophagy induced by D‐gal
JCR Q3 IF:5.7^21^	H9c2	20 g/L	72 h	Acacetin; (0.3, 1 or 3 μM); 72 h	SA‐β‐gal staining and activity of β‐galactosidase↑——↓; p53, p21↑——↓		Mitophagy: LC3II/LC3I↓——↑; PINK1 and Parkin↓——↑(0.3 μM↔); depolarization↑—— ↓(0.3 μM↔); sirt6↓——↑	Acacetin significantly inhibited in vitro cardiac senescence induced by D‐galactose via Sirt1‐mediated activation of the Sirt6/AMPK signalling pathway, thereby enhancing mitophagy and preserving mitochondrial function
JCR Q3 IF:4.4^65^	H9c2	10 g/L	24 h	Klotho; 0.1 μg/ml; 24 h	SA‐β‐gal staining↑——↓; p53, p21, and p16↑——↓	ROS↑——↓	Autophagy: LC3II/LC3I, Beclin1↓——↑; Apoptosis: Bax↑——↓, Bcl2↓——↑, caspase‐3↑——↓; rapid nuclear changes with heterogeneous intensity and chromatin condensation↑——↓ Cytotoxicity: cardiomyocytes size, extensive cytoplasmic vacuolation, and granular cells↑——↓; LDH↑——↓	Klotho reduced cardiomyocytes senescence induced by D‐gal
JCR Q2 IF:3.5^66^	Rat primary cardiomyocytes	10 g/L	48 h	——	SA‐β‐gal staining↑; p53↑		Cell viability↓	D‐gal increased ageing‐related markers and decreased cell viability
JCR Q3 IF:5.7^67^	H9c2	10 g/L	24 h	——	SA‐β‐gal staining↑; p53, P21↑	ROS↑		ROS/NLRP3 pathways contributed to the pathogenesis of cardiocytes ageing
JCR Q2 IF:6.5^68^	H9c2	40 g/L	48 h	Resveratrol；25, 50, 100 μM	SA‐β‐gal staining↑——↓; cardiomyocyte proliferation↓——↑; calcium concentrations↑—— ↓(25 μM↔)	ROS↑—— ↓(25 μM↔)	Mitochondrial dynamics: Mitochondrial Elongation↑—— ↓(25 μM↔) Mfn1, Mfn2, OPA1↔, Drp1↓——↑(25, 50 μM↔) Proapoptotic: Bcl‐2, BAX↔	Resveratrol alleviated cardiomyocytes ageing phenotype and ameliorated mitochondrial elongation via Drp1/Parkin/PINK1 Signalling in Senescent‐Like Cardiomyocytes induced by Carbonyl cyanide 3‐chlorophenylhydrazone
JCR Q4 IF:3^69^	H9c2	10 g/L	24 h	Adiponectin (An adiponectin‐overexpression plasmid was transfected into D‐gal‐treated H9c2 cells)	P16, P21↑——↓	ROS↑——↓; MDA↑——↓		Adiponectin protected against cardiomyocyte senescence induced by D‐gal via AdipoR1/APPL1 signalling, and it attenuated oxidative stress in senescent H9c2 cells by inhibiting the HO‐1/HMGB1 signalling pathway
JCR Q2 IF:4.6^70^	H9c2	10 g/L	24 h	CD38 knockdown; Nicotinamide dinucleotide (NAD+); 1 mM, 24 h	CD38 knockdown: SA‐β‐gal‐positive cells↑——↓; p16, p21↑——↓ NAD+:SA‐β‐gal‐positive cells↑——↓	CD38 knockdown: ROS↑——↓, NOX4↑——↓; the level of total protein acetylation↑——↓ NAD+: ROS↑——↓; MDA↑——↓; SOD2↓——↑; NOX4↑——↓		D‐gal increased cellular senescence and oxidative stress, that CD38 knockdown and NAD+ decreased cellular senescence and oxidative stress
JCR Q2 IF:5.9^72^	H9c2	50 g/L	48 h	Mitochondrial‐targeting antioxidant(MitoTEMPO); 1 μM; 24 h		ROS↑——↓	Cellular intracellular free Zn^2+^ ([Zn2^+^]_i_), [Zn2^+^]_i_ in mitochondria ([Zn2^+^]Mit)↑——↓, [Zn2^+^]i in S(E)R([Zn2^+^]SER)↓——↑; MMP↑——↓	Mitochondria‐Targeting Antioxidant provided Cardioprotection through regulation of cytosolic and mitochondrial Zn^2+^ Levels with re‐distribution of Zn^2+^‐transporters in aged rat cardiomyocytes

Abbreviations: ↑, indicators increased under the action of D‐galactose; ↓, indicators decreased under the action of D‐galactose; ↑——↓, indicators increased under the action of galactose and decreased under the intervention; ↓——↑, indicators decreased under the action of D‐galactose and increased under the intervention; ↔, there was no change in the indicators under D‐galactose or intervention; (↔), under the intervention treatment of this dose, the indicators did not reverse the change caused by D‐galactose.

## 8 EFFECTS OF PROTECTIVE INTERVENTIONS ON THE D‐GALACTOSE‐INDUCED CARDIAC AGEING

Numerous studies have reported that many interventions have protective effects against D‐galactose‐induced cardiac ageing in vivo and in vitro. These interventions regulate different signs of ageing. Here, we classify and summarize recent intervention measures from the last five years (Tables 1, 2, 3, 4, 5, 6).

First, cardiac ageing markers are common indicators in the study of cardiac ageing and are the main prognostic markers for measuring the efficacy of interventions. As shown in Table 1, many drugs restore the increase in D‐galactose‐induced markers of cardiac ageing, such as Kanglexin, CQ, Lico D, Thymoquinone, Curcumin, NaHS, AOS, 17β‐Estradiol, PSP, natural flavone acacetin, CDDO‐Im, resveratrol, calcitriol, AOF, *Lactobacillus plantarum* CQPC11, KSFY02, and NJAU‐01, AVP, PSLKDT, EFC, and Mangiferin. In addition to drug treatment, Bo‐Htay et al.^12^ treated rat with HBOT to reduce the number of senescent cells determined by SA‐β‐gal staining. Furthermore, Bei et al.^16^ found that miR‐21 knockout has a protective effect against D‐galactose‐induced cardiac alterations.

Second, cardiac oxidative stress is closely related to inflammation and is a critical indicator of the efficacy of interventions on ageing. Some drugs have been used to reduce cardiac oxidative stress and inflammation, including Mangiferin, PNPH, ITPL, AIMP, *Lactobacillus plantarum* NJAU‐01, ZH, CQ, NaHS, AOS, PSP, CDDO‐Im, AOF, resveratrol, calcitriol, and DO (Table 2). Interestingly, study has shown that 4% H2 inhalation or H2‐rich water drinking can reduce cardiac oxidative stress.^35^ As reported in the articles, HBOT has the same effect.^12^

Third, apoptosis has been increasingly studied in association with cardiac ageing. Some drugs, such as Thymoquinone, Curcumin, and CDDO‐Im, work through the mitochondria‐initiated intrinsic pathway and the death receptor‐stimulated extrinsic pathway (Table 3). HBOT also has protective effects on D‐galactose‐induced cardiomyocyte apoptosis.^12^ Exercise training has also been shown to restore insulin‐like growth factor‐I receptor‐mediated survival signalling in D‐galactose induced‐ageing rats to suppress cardiac apoptosis.^5^

Fourth, the relationship between autophagy and cardiac ageing is becoming increasingly clear with growing research. CQ, Lico D, AOS, and 17β‐Estradiol alleviate cardiac ageing by promoting autophagy (Table 3), and HBOT acts similarily.^12^

Fifth, medical treatments such as AOS, natural flavone acacetin, melatonin, and HBOT alleviated D‐galactose‐induced cardiac ageing by maintaining myocardial mitochondrial function and integrity (Table 4). As summarized in Table 5, cardiac morphology is essential for maintaining normal cardiac function. Medical treatments, such as Mangiferin, Kanglexin, ZH, AOF, AOS, PSP, resveratrol, and calcitriol, and exercise training, can resist D‐galactose‐induced cardiac remodelling (Table 5). Moreover, Mangiferin, Kanglexin, NaHS, ZH, AOS, 17β‐Estradiol, PSP, natural flavone acacetin, and AOF have been reported to have a positive effect on cardiac function (Table 5). Besides drug therapy, HBOT and miR‐21 knockouts also improve cardiac function.^12^, ^16^

As shown in Table 6, the interventional therapy of D‐galactose‐induced ageing is very similar in vitro and in vivo. Medical treatments, such as recombinant protein klotho, Nicotinamide dinucleotide (NAD+), mitochondrial‐targeting antioxidant(MitoTEMPO), Kanglexin, emodin, acacetin, and Resveratrol, reduce cardiac ageing markers, oxidative stress, and inflammation (MitoTEMPO has no effect on ageing markers, and acacetin has no effect on oxidative stress and inflammation) (Table 6). Additionally, CD38 knockdown and adiponectin‐overexpression plasmid was used in D‐gal‐treated H9c2 cells to play the same role.^69^, ^70^ Many studies have shown that klotho, Kanglexin, emodin, acacetin, Resveratrol, and MitoTEMPO have protective effects on D‐galactose‐induced cardiac ageing in vitro by altering apoptosis and Autophagy as well as cardiac mitochondrial function (Table 6).

## 9 CONCLUSIONS

This review mainly summarizes the literature on D‐galactose‐induced cardiac ageing in the last five years. D‐galactose induces cardiac ageing by increasing ageing‐related markers, upregulating oxidative stress and inflammation, altering autophagy and apoptosis of cardiomyocytes, remodelling cardiac morphology, and impairing cardiac function. We postulate that D‐galactose is a reliable for establishing ageing models for studying ageing‐related diseases and anti‐ageing therapeutic interventions.

## 10 LATEST ADVANCES

Although this review is not the first to summarize D‐galactose‐induced cardiac ageing (see article for details), herein, we discuss the recent advances in D‐galactose‐induced cardiac ageing research, which has rapidly increased in recent years. We believe that it is necessary to provide an updated review in this field, particularly recent advancements, including the indicators of cardiac function that are rarely discussed in previous reviews. First, the role of autophagy in D‐galactose‐induced cardiac ageing has been widely reported in recent studies. Table 3 summarizes the changes in autophagy after D‐galactose‐induced cardiac ageing. Table 5 describes the changes in cardiac morphology and cardiac function‐related indicators, which is a detailed and comprehensive improvement to previous reviews. Finally, recently, intervention options for D‐galactose‐induced cardiac ageing have become increasingly diverse. Therefore, we marked the protocol of intervention in each table, and summarized the efficacy of various intervention measures on different indicators at the end of the article for convenience.

## AUTHOR CONTRIBUTIONS

**Sui‐sui Wang:** Conceptualization (lead); data curation (lead); formal analysis (lead); investigation (lead); resources (lead); software (lead); writing – original draft (lead); writing – review and editing (lead). **Xu Zhang:** Data curation (equal); supervision (supporting); writing – review and editing (equal). **Ze‐zhi Ke:** Investigation (equal); writing – review and editing (supporting). **Xiu‐yun Wen:** Data curation (supporting); investigation (supporting). **Wei‐dong Li:** Data curation (supporting); writing – review and editing (supporting). **WenBin Liu:** Investigation (supporting); writing – review and editing (supporting). **Xiao‐dong Zhuang:** Resources (equal); writing – review and editing (equal). **Li‐zhen Liao:** Resources (equal); writing – review and editing (equal).

## CONFLICT OF INTERESTS

The authors declare no conflict of interest.

## Data Availability

Data openly available in a public repository that issues datasets with DOIs.
